# Ionic Crosslinked Hydrogel Films for Immediate Decontamination of Chemical Warfare Agents

**DOI:** 10.3390/gels10070428

**Published:** 2024-06-28

**Authors:** Gabriela Toader, Raluca-Elena Ginghina, Adriana Elena Bratu, Alice Ionela Podaru, Daniela Pulpea, Traian Rotariu, Ana Mihaela Gavrilă, Aurel Diacon

**Affiliations:** 1 Military Technical Academy ‘Ferdinand I’, 39-49 George Coșbuc Blvd., 050141 Bucharest, Romania; gabriela.toader@mta.ro (G.T.); podaru.alice04@gmail.com (A.I.P.); daniela.pulpea@mta.ro (D.P.); traian.rotariu@mta.ro (T.R.); 2 Research and Innovation Center for CBRN Defense and Ecology, 225 Olteniței Blvd., 077160 Bucharest, Romania; raluca.ginghina@nbce.ro (R.-E.G.); adriana.bratu@nbce.ro (A.E.B.); 3 Faculty of Chemical Engineering and Biotechnologies, National University of Science and Technology Politehnica of Bucharest, 1-7 Gh. Polizu Street, 011061 Bucharest, Romania; 4 National Institute of Research and Development for Chemistry and Petrochemistry, 202 Splaiul Independentei, 060041 Bucharest, Romania; ana.gavrila@icechim.ro

**Keywords:** hydrogels, mustard gas decontamination, photocatalytic degradation, mechanical properties of hydrogels, ionic crosslinking

## Abstract

This study describes the development of hydrogel formulations with ionic crosslinking capacity and photocatalytic characteristics. The objective of this research is to provide an effective, accessible, “green”, and facile route for the decontamination of chemical warfare agents (CWAs, namely the blistering agent—mustard gas/sulfur mustard (HD)) from contaminated surfaces, by decomposition and entrapment of CWAs and their degradation products inside the hydrogel films generated “on-site”. The decontamination of the notorious warfare agent HD was successfully achieved through a dual hydrolytic–photocatalytic degradation process. Subsequently, the post-decontamination residues were encapsulated within a hydrogel membrane film produced via an ionic crosslinking mechanism. Polyvinyl alcohol (PVA) and sodium alginate (ALG) are the primary constituents of the decontaminating formulations. These polymeric components were chosen for this application due to their cost-effectiveness, versatility, and their ability to form hydrogen bonds, facilitating hydrogel formation. In the presence of divalent metallic ions, ALG undergoes ionic crosslinking, resulting in rapid gelation. This facilitated prompt PVA-ALG film curing and allowed for immediate decontamination of targeted surfaces. Additionally, bentonite nanoclay, titanium nanoparticles, and a tetrasulfonated nickel phthalocyanine (NiPc) derivative were incorporated into the formulations to enhance absorption capacity, improve mechanical properties, and confer photocatalytic activity to the hydrogels obtained via Zn^2+^—mediated ionic crosslinking. The resulting hydrogels underwent characterization using a variety of analytical techniques, including scanning electron microscopy (SEM), Fourier transform infrared spectroscopy (FTIR), viscometry, and mechanical analysis (shear, tensile, and compression tests), as well as swelling investigations, to establish the optimal formulations for CWA decontamination applications. The introduction of the fillers led to an increase in the maximum strain up to 0.14 MPa (maximum tensile resistance) and 0.39 MPa (maximum compressive stress). The UV-Vis characterization of the hydrogels allowed the determination of the band-gap value and absorption domain. A gas chromatography–mass spectrometry assay was employed to evaluate the decontamination efficacy for a chemical warfare agent (sulfur mustard—HD) and confirmed that the ionic crosslinked hydrogel films achieved decontamination efficiencies of up to 92.3%. Furthermore, the presence of the photocatalytic species can facilitate the degradation of up to 90% of the HD removed from the surface and entrapped inside the hydrogel matrix, which renders the post-decontamination residue significantly less dangerous.

## 1. Introduction

Non-proliferation and disarmament efforts can play a crucial role in countering terrorism by preventing or reducing the access of non-state actors or unauthorized individuals to chemical, biological, and nuclear dual-use materials [1]. The illicit proliferation of chemical weapons, clandestine production of toxins and biological agents, the development of ‘dirty bombs’, and the trafficking of fissile material are just a few examples of how CBRN agents can be exploited for terrorist purposes. In the event of a chemical, biological, radiological, and nuclear (CBRN) attack or accidental release of hazardous materials, both military personnel and civilians may face contamination situations. Trained first responders will promptly initiate decontamination procedures, in strict adherence to CBRN incident regulations, with the aim of restoring safety to the contaminated site. Their immediate response is crucial to minimizing the penetration of the hazardous agent through protective/non-protective equipment and surfaces, thereby preventing significant casualties [2]. The actions taken within the first 10 min following a CBRN incident can significantly influence the outcome of the situation [2].

In certain CBRN scenarios, it may be necessary to implement decontamination principles, procedures, and methods in a distinct manner. Among all CBRN agents, chemical warfare agents (CWAs) are particularly lethal and insidious, presenting a significant challenge for decontamination efforts. Moreover, only a few chemical warfare agents have effective antidotes. In this scenario, it is of utmost importance to prioritize the implementation of safety protocols for injured individuals as the primary life-saving measure. When conducting immediate decontamination procedures, it is important to thoroughly remove any accessible traces/droplets that may contain chemical warfare agents. Every contamination trace requires diligent attention. The decontamination of chemical warfare agents and their simulants is also crucial in research laboratories or CWA destruction sites to mitigate the threat posed by chemical warfare weapons [3]. Numerous military organizations have established detailed standard operating procedures and decontamination formulations for rapid decontamination, but there is still significant potential for improvement in effectively addressing the environmental impact of these formulations. Certain defense forces utilize Fuller’s earth [4] as a solid absorbent for chemical warfare agents. This absorbent, based on clay material, demonstrates the capability to absorb liquid contaminants, such as CWAs, and effectively facilitate their removal from surfaces or individual equipment. Dry decontaminants such as Fuller’s earth have a significant drawback in that they do not degrade or detoxify hazardous chemical agents. Consequently, the sorbent material must be meticulously collected to prevent toxic fine dust or particulates from being inhaled or adhering to exposed skin during desorption [4,5]. Alkaline solutions, oxidizers, or bleaching formulations utilized for the decontamination of CWAs may entail potential risks to human health due to the presence of volatile solvents and corrosive components [6]. Addressing these potential risks is important to ensure the safety of individuals involved in the decontamination process.

Aqueous decontaminants have been extensively utilized by military entities worldwide and have been recognized as a cost-effective decontamination method since World War I. However, it is important to acknowledge their limitations, especially when dealing with persistent chemical warfare agents like sulfur mustard [7,8]. A chemical warfare agent is considered to be persistent when contamination lasts over a minimum of 24 h [2]. According to this definition, sulfur mustard (HD) is a very persistent blistering chemical warfare agent. In an aqueous environment, the hydrolysis rate of HD is relatively low [9], which presents challenges for decontamination processes using plain water. Thus, decontamination of HD-contaminated surfaces with plain water, and even with water plus detergents, may not yield optimal results [10]. In recent years, several studies have highlighted the development of water-based peelable formulations intended to serve as environmentally friendly solutions [11,12] for the decontamination of CWAs. This is in response to the challenges posed by current decontamination procedures, which are labor- and resource-intensive, require excessive water usage, and may involve corrosive or toxic elements, presenting environmental concerns [13].

Peelable coatings offer several advantages over formulations based on volatile solvents. These include a simpler process (applying the film-forming solution/gel and then peeling the resulting membrane after complete curing), likely inactivation and entrapment of contaminants, reduced post-decontamination waste, and the advantage of using safe aqueous-based polymeric formulations. Gel-based membranes have recently attracted attention not just for separation but also for additional functions such as sensing, antimicrobial activity, catalysis, or decontamination applications [11,14,15,16]. Most gel-based membranes consist of a polymeric backbone, which can be natural polymers like agarose, chitosan, and sodium alginate, or synthetic polymers like polyvinyl alcohol (PVA), polyacrylamide (PAM), and polyethylene glycol (PEG) [14,17]. Because of the considerable water content, these membranes generally exist in a swollen state, where water molecules are enclosed within the polymeric matrix [14]. Crosslinking agents are frequently utilized in gel-based membranes to optimize their stability and regulate their mechanical properties [14,15,18]. The design of gel-based membranes can be modulated through the variation of crosslinking density, resulting in improved mechanical strength and the ability to tailor swelling characteristics. Increasing the crosslinking density usually results in a stiffer material that is less prone to swelling [14].

Incorporating supplementary active ingredients, such as photoactive compounds, can enhance gel-based formulations with catalytic, photocatalytic, or decontaminating properties. When combined with specific nanoparticles or dyes, gel-based membranes can exhibit adjustable optical properties that are valuable in applications such as sensing or decontamination [14]. Phthalocyanines are macrocyclic compounds derived from the basic structure of porphyrins. They are typically used in photodynamic therapy, catalysis, and photocatalysis, as well as in the dye industry. Due to their low toxicity and strong efficacy in oxidizing common organic contaminants, photo-induced reactions involving phthalocyanines hold promise for CWA decontamination applications [19,20].

Recent studies have documented the testing of various types of peelable membranes/films designed to collect and degrade pesticides and chemicals associated with chemical warfare agents on a variety of surfaces, including metal, upholstery, flooring, plastic, drywall, textiles, and glass. These polymeric-based films are intended for sampling purposes [21], as a protective barrier against CWAs [22], or for the decontamination of chemical warfare agents [11,15,23,24,25]. Bret A. Voss et al. described gel-based coatings designed as a barrier and decontaminating formulation for chloroethylethylsulfide (CEES) [26,27], a simulant for mustard gas vapors. In our previous studies, we described various types of film-forming decontaminating formulations obtained with the casting method [11], photopolymerization [15], or ionic crosslinking [28], specially designed for the decontamination of CBRN agents. Nevertheless, the recent formulations may benefit from further enhancements in terms of curing time, environmental friendliness, and cost-effectiveness. Ionic crosslinked gels can offer superior performance but also efficient and rapid scale-up of the film-formation step [28].

In this context, this new study presents a straightforward CWA decontamination approach by entrapping the chemical warfare agents inside a polymeric peelable coating by utilizing “green” film-forming ingredients (such as sodium alginate and polyvinyl alcohol, incorporating absorbent components such as bentonite nanoclay and/or photocatalyst such as titanium dioxide, a water-soluble sulfonated nickel phthalocyanine derivative). The gel-based formulation is designed for direct application onto contaminated surfaces. It utilizes Zn^2+^ as an ionic component to obtain rapid and efficient on-site crosslinking of the membrane, thereby enabling fast and effective entrapment of the liquid or vapor phase of persistent chemical warfare agents (CWAs) such as sulfur mustard (HD). The considerable absorptive capacity of bentonite nanoclay [29,30], combined with the cumulative photocatalytic activity of TiO_2_ [31,32] and phthalocyanine [19,33,34], also ensures the concurrent degradation of the contaminant and its sequestration within the polymeric network of the hydrogel film. This method significantly reduces curing time, via ionic crosslinking of the polymeric membrane, offering a faster and more efficient CWA decontamination tool.

## 2. Results and Discussion

### 2.1. Method Principles

The aim of this study is to ascertain the possibility of employing readily available ionic crosslinkable hydrogel polyvinyl alcohol–alginate films for the decontamination of chemical warfare agents from various types of surfaces (e.g., working benches in specialized laboratories, sites dedicated to CWA destruction, sensitive equipment, etc.). The decontamination formulations also incorporate bentonite, titanium dioxide nanoparticles, and tetra-sulfonated nickel phthalocyanine. Their influence on the final properties of the hydrogel films and the corresponding variations in decontamination efficacy were subjected to further comprehensive evaluation. The proposed strategy aims to facilitate the rapid formation of hydrogel films from aqueous solutions. These films are designed to effectively remove and entrap the chemical warfare agents from contaminated surfaces, as well as neutralize the CWAs captured inside the films once they are removed from the surface.

**Scheme 1:**
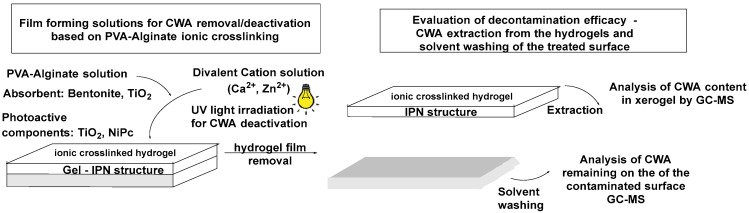
Objectives of this study and basic principles.

Scheme 1 illustrates the basic principles of this decontamination method. It implies the use of PVA–alginate-based hydrogels incorporating an adsorbent (bentonite), titanium dioxide, and a tetrasulfonated nickel phthalocyanine derivative (NiPc) designed for CWA removal and deactivation from a contaminated surface. In this experimental study, we focused on studying the ability of the designed formulations to generate a nanocomposite semi-interpenetrating polymer network (semi-IPN [35]), via ionically driven crosslinking of ALG in the presence of PVA, a phthalocyanine derivative, and the nanofillers (bentonite nanoclay and TiO_2_ nanoparticles) and their ability to degrade HD and to entrap post-decontamination residual products.

### 2.2. Morphological Characterization of the Hydrogels

The dried hydrogels were investigated via SEM analysis and EDX spectroscopy to evaluate their morphology and elemental composition. From the images presented in the Supplementary Materials file, we can observe a modification of the pore formation between the samples (as highlighted for Alg-1–Alg-5 in Figure S1). Compared to the blank sample Alg-1, the introduction of the bentonite in Alg-2 leads to the formation of a smaller number of larger pores which can be explained by the interaction between bentonite and the OH of the polymer matrix. However, the combination of bentonite and TiO_2_ lead to a reduction in pore density and size. The films containing NiPc exhibited a smoother surface, with minimal formation of small pores. The absence of large pores in the membrane films containing NiPc (Alg-4c1, Alg-4c2, and Alg-5) can be advantageous for storage and disposal following the decontamination process. The presence of numerous large pores could be problematic, potentially leading to the accidental release of trapped contaminants during hydrogel storage. The EDX spectra of the obtained hydrogel films are in agreement with the sample composition, confirming the presence of the clay (bentonite) (Al, Si), TiO_2_ (Ti), or phthalocyanine derivative (Ni) in addition to the Zn used for the ionic crosslinking and C and O present in the polymer matrix. The elemental mapping analysis conducted for sample Alg-5 (Figure S2) has confirmed a uniform distribution of all components within the nanocomposite semi-IPN films. Additionally, it has indicated the presence and even distribution of zinc ions, which were used in the crosslinking process.

### 2.3. Swelling Degree

**Figure 1:**
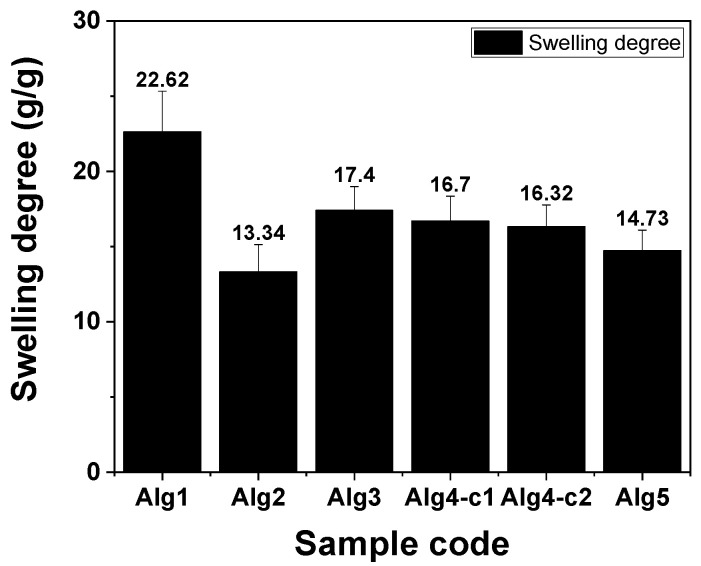
Swelling degree obtained for the selected ionic crosslinked hydrogel formulations.

The hydrogel formulations developed for this study were analyzed to determine their swelling degree (the ability to absorb water) (Figure 1). The highest swelling capacity can be noticed for Alg-1 (reference sample, which does not contain any other supplementary component). The difference in swelling capacity may be attributed to the enhanced interaction of the polymer components with water molecules. Conversely, the incorporation of adsorber components (bentonite) or photoactive components (TiO_2_ or Pc) in the other samples results in a diminished swelling capacity. This decrease is a consequence of the hydroxyl groups of the polymer components (PVA and alginate) interacting with the other components. The lowest swelling capacity was registered for hydrogels containing only bentonite (Alg-2). In the case of sample Alg-5, which encompasses all the components (including bentonite, TiO_2_, and Pc), the swelling capacity exhibits a slight increase compared to Alg-2. This phenomenon can be attributed to the adsorption of TiO_2_ and phthalocyanine by bentonite, consequently diminishing their interaction with the polymer matrix.

### 2.4. FTIR Analysis

**Figure 2:**
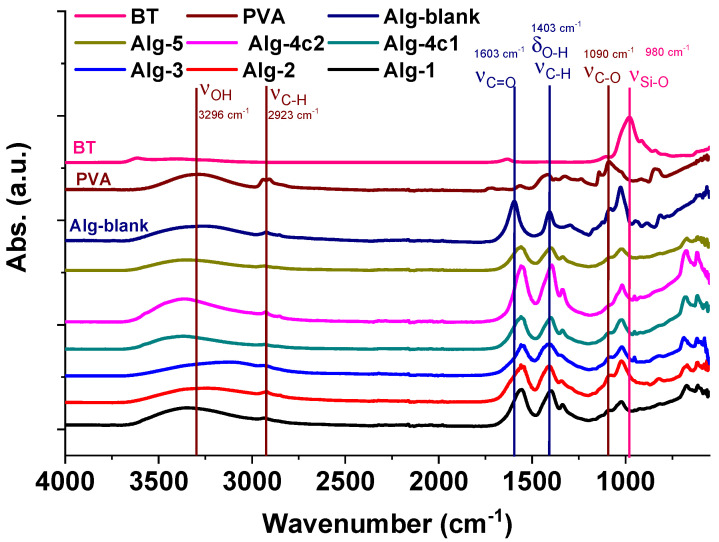
FTIR spectra of the hydrogels and reference PVA, alginate (alginate blank), and bentonite (BT).

FTIR analysis was utilized to investigate the obtained hydrogels to ascertain their composition and the interactions established inside the crosslinked structures specifically designed for decontamination applications. FTIR analysis (Figure 2) revealed the corresponding adsorption bands for the six formulations tested in this study. The presence of a wide absorption band between 3400 and 3100 cm^−1^ can be attributed to the ν_O-H_ or ν_N-H_ stretching vibrations. There is a difference between the maximum of this band as compared to the blank (PVA and alginate) samples. This modification of the FTIR absorption bands can sustain the explanation for the swelling degree modification due to the introduction of bentonite or TiO_2_. Thus, a modification of the O-H vibration caused by the polymer matrix interacting with the added fillers can account for the lower swelling degree compared to the blank sample (Figure 1). The second absorption band present at around 2930 cm^−1^ can be attributed to ν_C–H_ stretching vibrations. The absorption band at 1603 cm^−1^ could be assigned to ν_C=O_ stretching from the carboxyl group of the alginate and esters in the non-hydrolyzed PVA. Compared to the alginate sample, all formulations display a shift of the maximum for C=O stretching vibration, confirming the formation of different hydrogen bond interactions. The presence of carboxylic groups on the polymer chains is also confirmed by the appearance of the peak situated at δ_O–H_ 1403 cm^−1^, characteristic of the bending vibrations of the O–H bond from the carboxylic acids. Also, the δ_C–H_ bending vibrations are visible at 1430 cm^−1^. The adsorption band characteristic for C–O stretching vibrations is represented through the ν_C–O_ at 1090 cm^−1^, asymmetric stretching. Bentonite nanoclay presence in the samples is confirmed with the Si–O–Si vibration band at 980 cm^−1^.

### 2.5. Mechanical Properties of the Hydrogels

**Figure 3:**
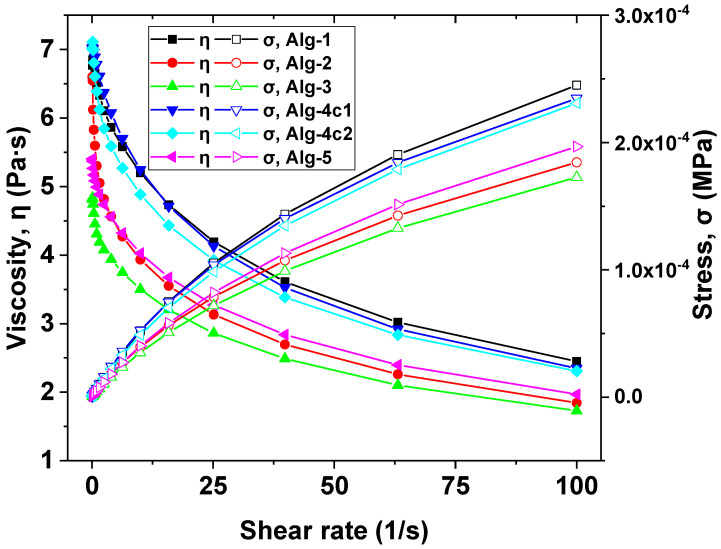
Viscosity analysis for the polymeric solutions prior to the addition of the ionic crosslinking agent.

An essential consideration in formulating a solution is the efficient dispersion of various components within the polymeric aqueous solution. Consequently, rheology analysis was conducted on the formulations prior to the ionic crosslinking process. The rheological analysis elucidates the relationship between shear rate and stress response of the solutions. All formulations demonstrated shear-thinning behavior, characterized by a decrease in viscosity as the shear rate increased (Figure 3). The rheological analysis highlights that the initial viscosity increases with the inclusion of bentonite, TiO_2_, and NiPc components, which could have practical implications when applying the solution to a contaminated area. Alg-3 exhibits the lowest viscosity due to the presence of bentonite with a lamellar structure and spherical titanium nanoparticles, probably leading to a distinct rearrangement of the polymer chains.

**Figure 4:**
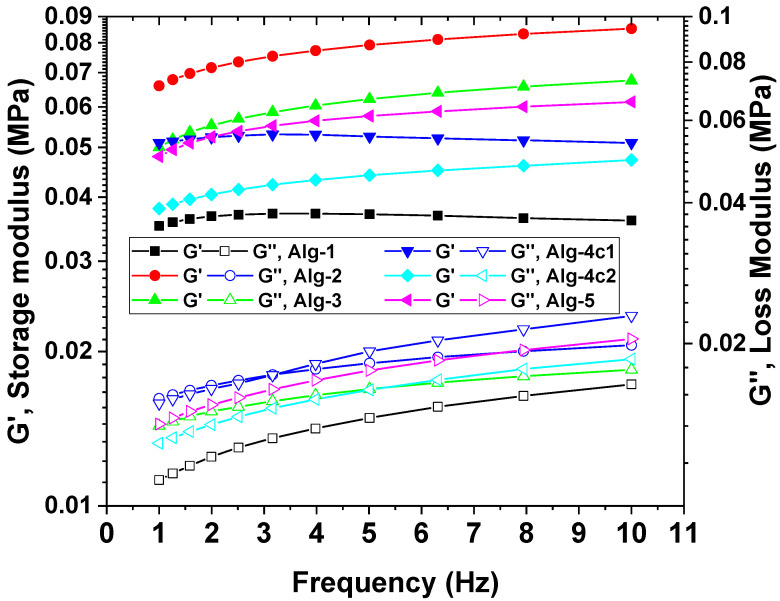
Frequency-dependent shear tests for the crosslinked hydrogels—storage modulus and loss modulus plots.

The next step involves evaluating the viscoelastic properties of the crosslinked hydrogels. The behavior of the films can provide insight into the suitability of the gels for decontamination purposes. Specifically, the information obtained can be further correlated with their adhesion to the surface and their response during the peeling process. Frequency-dependent shear tests, as illustrated in Figure 4, demonstrated an increase in the loss modulus (G″) with frequency. Crosslinking results in a higher elastic modulus compared to the loss modulus, with minimal frequency dependency. A high degree of crosslinking leads to an increase in the loss modulus, suggesting energy dissipation during bond rearrangement [36]. Increased levels of crosslinking are usually associated with a greater dissipation of energy and a higher loss modulus.

The entanglements of the polymer chains may also function as supplementary crosslinks, potentially impeding excessive swelling [37]. In contrast to permanent chemical crosslinks, these entanglements exhibit flexibility and the capacity to transfer stress between chains effectively, thereby conferring elasticity and toughness to the hydrogel [37]. Except for Alg-1 and Alg-4c1, the storage modulus G’ shows a more pronounced increase with frequency. This suggests that the fillers generally have a stiffening effect, apart from Alg-1 (which does not contain fillers) and Alg-4c1, where the presence of phthalocyanine at low concentrations can disrupt some intermolecular interactions. In the latter case, the storage modulus increases but then levels off. Hence, it can be concluded that the increase in frequency, resulting in a higher storage modulus, signifies a prevalence of elastic behavior, while the viscous characteristic is diminished [38,39]. G’ > G″ may also be indicative of a high elastic behavior of the ionically crosslinked hydrogels.

To achieve efficient decontamination of the targeted areas, alongside good adsorption characteristics, the hydrogels should possess good tensile strength and mechanical resistance to facilitate complete removal from the surface and limit cross-contamination during disposal of the waste material.

**Figure 5:**
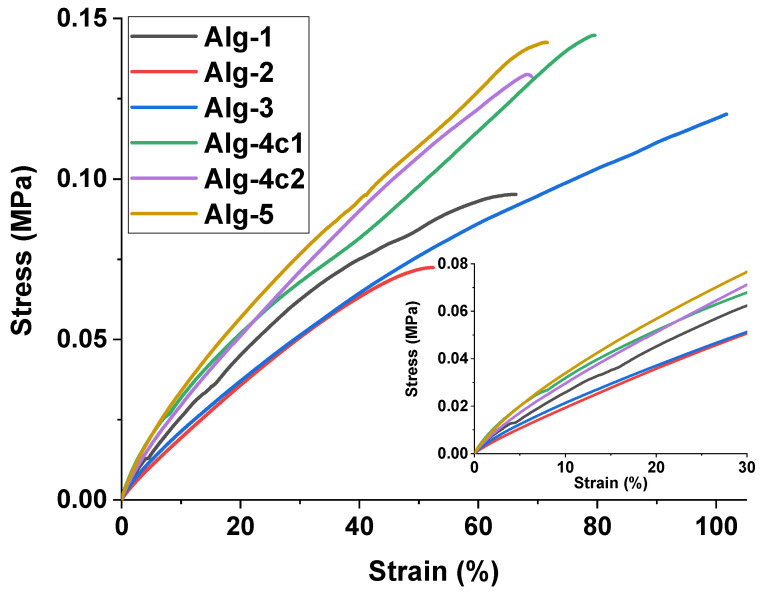
Tensile stress–strain curves of the obtained hydrogels.

The tensile strength test (Figure 5 and Table 1), conducted after the completion of ionic crosslinking, indicated that the presence of the nanofillers resulted in enhanced mechanical resistance for all samples except for Alg-2. The combination of TiO_2_ nanoparticles with bentonite nanoclay led to the largest increase in the maximum strain (Alg-3). The presence of NiPc has a noticeable impact on the tensile strength. In the case of Alg-5, which encompasses all the components, the maximum stress ranks as the second highest among all six formulations, with the maximum stress exceeding 70%. The variations in mechanical properties could potentially be attributed to the formation of hydrogen bonds between the polymer matrix and the additional fillers.

**Figure 6:**
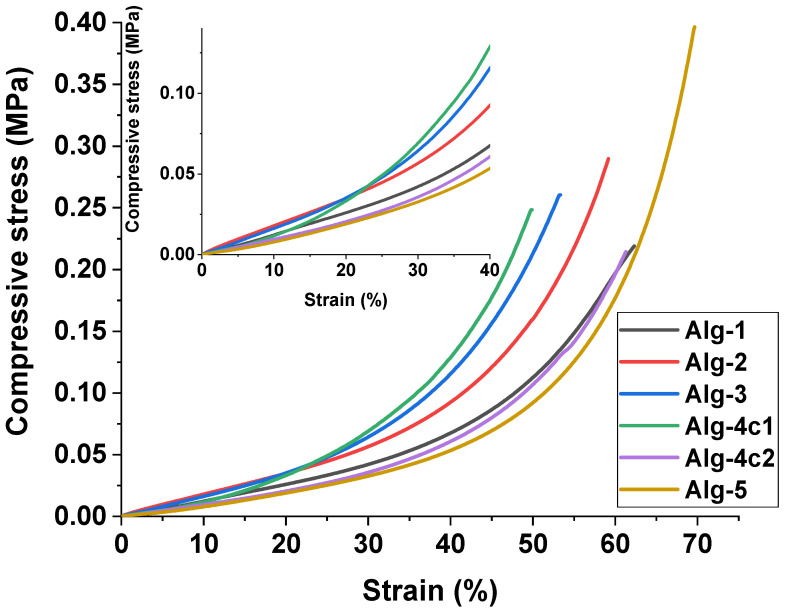
Stress–strain curves of the obtained hydrogels.

The uniaxial compression tests (Figure 6 and Table 2), performed on the hydrogels in a fully swollen state, revealed that the addition of the fillers/active components to the Alg-1 formulation overall increases the mechanical characteristics of the gels. The highest maximum stress resistance and maximum strain were obtained for sample Alg-5 (which contains all the components). It is worth noting that at lower strain values (20%), samples Alg-4c2 and Alg-5 demonstrate lower resistance compared to the blank Alg-1, suggesting increased malleability. However, it is important to acknowledge that, ultimately, all samples exhibited stress values comparable to or higher than Alg-1. The lowest maximum strain was registered for Alg-3, which contains both bentonite nanoclay and TiO_2_ nanoparticles, and for Alg-4c1. In both instances, the fillers have the capacity to form hydrogen bonds with the polymer matrix. However, the presence of nanofillers also leads to a higher maximum stress. Conversely, at lower concentrations, the inclusion of phthalocyanine does not demonstrate an improvement in compressive stress resistance, resembling the observed alteration in tensile strength as illustrated in Figure 5 for Alg-4c1. The compressive values measured could be considered adequate to enable the safe handling of the waste material following decontamination, thus helping to minimize the potential for cross-contamination or inadvertent release of the entrapped CWA agent.

### 2.6. Decontamination Survey

**Figure 7:**
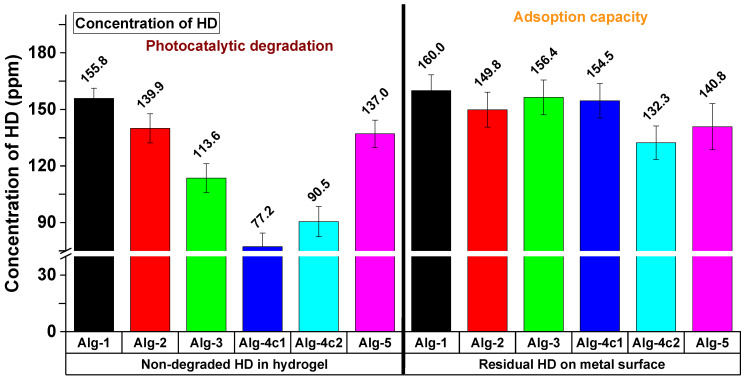
HD concentration determined via GC-MS post-decontamination survey on the extracted hydrogel (assessment of photocatalytic degradation abilities) and residual HD found on the decontaminated metal surface (assessment of adsorption capacity of the formulations).

Our previous study confirmed that CWA decontamination can be enhanced by UV irradiation on targeted areas when synergistic hydrolysis and photocatalytic processes take place during the photopolymerization of monomer species and film formation [15]. In the current decontamination scenario, the film formation involves ionic crosslinking, and thus, the number and/or type of radical species generated will be dictated by the photoactive components in the hydrogels. Figure 7 illustrates the outcomes of post-decontamination, specifically the concentration of residual HD on the contaminated metal surface and the presence of undegraded CWA in the hydrogel. The results show that the best adsorption characteristics of the hydrogel were registered for Alg-4c2, followed by Alg-5. Thus, all five hydrogel formulations displayed superior absorption characteristics compared to the blank sample Alg-1. Particularly, the presence of NiPc proved beneficial for HD removal from the metal surface. In terms of HD degradation, the best results were observed for Alg-4c1 and Alg-4c2. All five hydrogel formulations displayed lower residual HD concentration compared to Alg-1. The decreased HD degradation performance observed in Alg-4c2, in comparison to Alg-4c1, can be attributed to the higher concentration of NiPc, which may hinder efficient photocatalytic activity, likely due to the ineffective excitation of the dye molecules. Also, in the case of Alg-5, the presence of TiO_2_ also decreases the transmittance of the hydrogels, resulting in a lower penetration of light and, thus, a less efficient generation of radical species.

**Figure 8:**
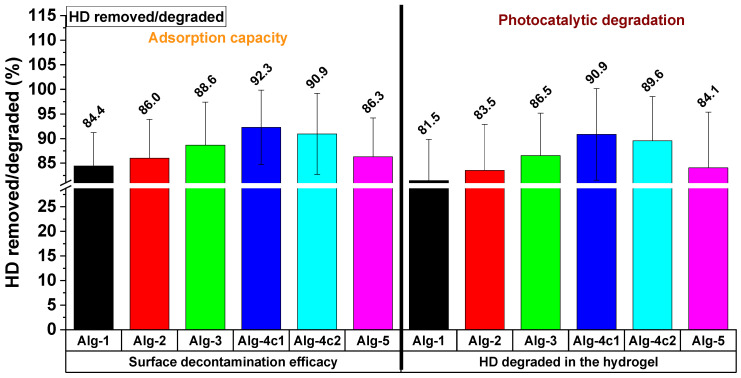
Decontamination efficacy evaluated with GC-MS for establishing the difference between the initial contamination level and the amount of degraded HD found in the hydrogel and the residual HD found on the targeted metal surface after the completion of the decontamination process.

Figure 8 illustrates the overall effectiveness of the developed hydrogel formulations in decontaminating HD, showing the percentage of HD degraded from the amount entrapped in the films removed from the surface. Thus, sample Alg-4c1 achieved the highest decontamination efficacy (92.3%), surpassing the blank sample Alg-1 (84.4%). Also, in terms of degraded sulfur mustard found inside the hydrogel membrane, Alg-4c1 also displays the highest degradation capacity. The employment of NiPc yields two distinct advantages in the field of decontamination: higher absorption capacity and augmented HD degradation at reasonably low concentrations. Additionally, the incorporation of NiPc into the hydrogel formulation enhances the mechanical properties of the films to a certain extent. Therefore, the presence of the NiPc derivative coupled with the use of an ionic crosslinking strategy offers an innovative, fast, reliable strategy for HD removal from contaminated surfaces and the generation of less dangerous post-decontamination residues/materials. The UV-Vis absorption characteristics of NiPc allow for the potential reduction in toxicity in waste materials with the assistance of solar light. The UV-Vis spectra and the Tauc plots presented in Figure S6A–C allow the evaluation of the optical characteristics of the hydrogel films and of the band-gap of the photocatalytic components [40,41]. Thus, TiO_2_ displayed a band-gap value of 3.53 eV. For the phthalocyanine-containing samples, the UV-Vis spectra depict the absorption bands in the UV region (B band—specific for the π-π*) followed by the specific Q band in the visible region [42]. Thus, a slight modification of the Q band is observed at higher NiPc concentration in the hydrogel Alg-4c2. Furthermore, the presence of NiPc reduces the band-gap compared to only TiO_2_ and enhances the photocatalytic activity of the materials, allowing also the utilization of the visible light spectrum for the photocatalytic degradation process, which could be performed using solar spectrum light at a larger scale.

A comparison of our decontamination and deactivation results with previous literature examples was performed in Table 3.

In our previous studies [11,15], we studied peelable coatings obtained via simple air-drying methods and photopolymerization, which resulted in similar or slightly higher decontamination efficacies for HD (but with higher resource consumption, such as longer curing times or higher costs for the monomers). When comparing the current results with other similar studies (Table 3) or, for example, those conducted by Marek Andrle et al. [19] (who obtained a decomposition efficacy of yperite (HD) in methanol that did not exceed 80% even after 7 h when using a phthalocyanine derivative embedded in an ethylene–vinyl acetate polymer matrix), we can conclude that the herein described method provides optimal results in terms of eco-friendliness, costs, and decontamination efficiency for HD (decontamination efficacies >90% as required by NATO standards [48]). A general HD degradation pathway mechanism [49] that involves both hydrolytic steps (facilitated by the water present in the hydrogels) and reactive superoxide radical anions (O_2_∙^−^) species and/or hydroxyl radical (HO∙) species generated due to the presence of the photocatalytic species is presented in Figure S8.

## 3. Conclusions

This study focused on developing an efficient strategy for CWA decontamination and degradation using hydrogel coatings that are ionically crosslinked and display photocatalytic activity. To enhance both the adsorption characteristics and photocatalytic activity, the developed formulations incorporate bentonite nanoclay, TiO_2_ nanoparticles, and/or a NiPc derivative. The investigations focused on the mechanical properties and decontamination characteristics of the hydrogels, aiming to establish correlations with the composition of the decontaminating formulations. Thus, the mechanical properties assessment revealed an increase in tensile strength resistance and compression strength through the introduction of the nanofillers and/or NiPc, which can be attributed to the formation of hydrogen bond interactions between the polymer matrix (polyvinyl alcohol and alginate) and the fillers. The decontamination experiments assisted with GC-MS resulted in HD removal efficiencies of over 90%, along with a degradation efficacy of over 90% of the HD sequestered in the polymeric films. When evaluating the optimal formulation, it is important to consider both the efficacy of HD removal and its deactivation within the hydrogel. Thus, a higher phthalocyanine concentration and/or presence of TiO_2_ lead to a decrease in the degradation process by limiting the light penetration through the gel. Our best formulation was Alg-4c1 which offered adequate mechanical properties coupled with 92.3% HD removal efficiency and 90.9% degradation efficacy inside the hydrogel.

The progress achieved in this experimental study, coupled with the simplicity and cost-effectiveness of these eco-friendly decontamination formulations, can represent a noteworthy advancement in the field of decontamination applications.

## 4. Materials and Methods

### 4.1. Materials

The materials employed in the synthesis process and used as received included the following: polyvinyl alcohol (PVA, MW 85,000–124,000, 87–89% hydrolyzed Sigma-Aldrich, St. Louis, MO, USA), hydrophilic bentonite nanoclay (BT, Sigma-Aldrich, St. Louis, MO, USA), sodium alginate (ALG, from brown algae, Carl Roth, Karlsruhe, Germany, MW 300,000–350,000 g/mol), nickel(II) phthalocyanine-tetrasulfonic acid tetrasodium salt (NiPc, Sigma-Aldrich, St. Louis, MO, USA), and zinc acetate dihydrate (Zn(CH_3_COO)_2_2H_2_O, Sigma-Aldrich, St. Louis, MO, USA). TiO_2_ nanoparticles were synthesized according to literature procedures [50] and characterized in our previous study [51], they consist of nanoparticles with mostly spherical shapes with a mean diameter of 132 nm.

For the decontamination tests, real CWA was used: bis(2-chloroethyl) sulfide (HD, sulfur mustard, purity: 95%, own synthesis). All the tests implying the decontamination of the toxic agents utilized in this study were performed at the “Research and Innovation Center for CBRN Defense and Ecology”, in the “Chemical Analysis Laboratory”, an OPCW-designated laboratory.

### 4.2. Methods

#### 4.2.1. Formulation and Application of the Hydrogels

Table 4 illustrates the components of the decontaminating formulations utilized in this experimental study.

**Figure 9:**
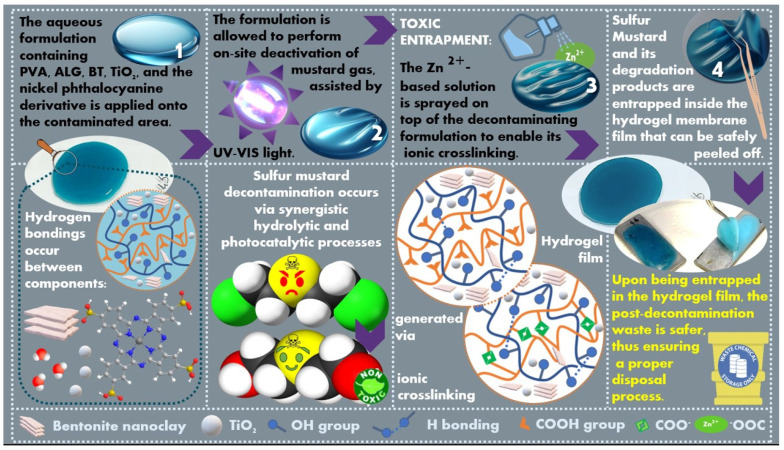
Method principle.

The graphical representation in Figure 9 illustrates the essential principles of the innovative decontamination method proposed in this study. As can be observed from this scheme (Figure 9); after applying the formulation on the contaminated site (step 1), the solution is allowed for 15 min to perform the ‘on-site’ deactivation of sulfur mustard, assisted with UV-Vis light (step 2). Subsequently, ionic crosslinking was achieved by spraying on top of the decontaminating formulation a water-based solution of zinc acetate (10 wt.%). The generation of the nanocomposite semi-IPN hydrogel film on-site involved the establishment of ionic crosslinkages (step 3). This was achieved by forming ionic bonds between ALG units and Zn^2+^ ions to ensure the formation of a 3D matrix. Prior to removal from the decontaminated surface, the nanocomposite hydrogel membrane films were permitted to remain in place for an additional 20 min. This extension ensured complete curing and maximal entrapment of any residual contaminants. Consequently, the exfoliated hydrogel films incorporating the post-decontamination residues can be safely disposed of (step 4).

The decontamination survey on the chemical warfare agent, sulfur mustard (HD), was meticulously evaluated in highly secure and controlled environments at an OPCW-designated laboratory from the Research and Innovation Center for CBRN Defense and Ecology, Bucharest, Romania. The procedures for controlled contamination and subsequent decontamination were conducted in accordance with the methods utilized in our prior studies [11,15]. This rigorous assessment strictly followed stringent safety protocols to ensure the utmost precaution and accuracy [48].

#### 4.2.2. Characterization

The FTIR analysis of the dried hydrogels and components was performed on a Spectrum Two FTIR spectrometer (PerkinElmer, Waltham, MA, USA) equipped with MIRacleTM Single Reflection ATR-PIKE Technologies, at 4 cm^−1^ resolution, totaling 32 scans, and 4000–600 cm^−1^ wavenumber range.

The swelling ability of the hydrogels (Equation (1)) was assessed according to refs. [15,52,53]. Xerogels ($w_{x}$) were weighted and were subsequently immersed and maintained (at room temperature) in distilled water, until they reached a constant weight ($w_{h}$).

(1) $$Swelling\;degree\left(g/g\right)=\frac{w_{h}-w_{x}}{w_{x}}$$

where $w_{h}$(g) represents the weight of the hydrogel in the equilibrium swollen state, and $w_{x}$ (g) is the weight of the completely dried xerogel.

The surface and the section morpho-structural characterization of the membranes were acquired with a field emission scanning electron microscope (SEM), equipped with Energy Dispersive X-ray Analysis (EDX), at an acceleration voltage of 15 kV. The backscattered electron (BSE) detector was used for all micrographs. For the morphological characterization of the membranes, a Hitachi TM4000 plus II microscope (Hitachi, Tokyo, Japan), with a cooling stage was used. Before analysis, the membranes were sputter-coated with a 5 nm thick gold layer using a Sputter Coater Q150R ES Plus instrument (Quorum Technologies, SXE, Lewes, UK). The elemental mapping was assessed with the EDX technique (EDS Detector AZtecONE, OXFORD Instruments, High Wycombe, UK). EDX analysis was performed at the following operational parameters: SEM magnification 100×, applied voltage of 15 keV, working distance of 9.61 mm, pixel of 500 ms, process time sensitive, and a live counting time of 20 s. Elemental mapping and spectrum quantification were performed by means of the AZtecOne 4.3 software. The mechanical characteristics of the obtained hydrogels were assessed using a DMA 850 instrument from TA Instruments, utilizing specific clamps for each type of test, as follows: the tensile clamp was utilized for subjecting five rectangular hydrogel specimens from each type of sample to the uniaxial tensile test, performed at 5 mm/min, in rate control–strain ramp mode, and the mean values were reported; the compression clamps (Ø40 mm) were utilized for subjecting five fully swollen disc specimens from each sample to uniaxial compressive loading at 2 mm/min, in rate control–strain ramp mode, and mean values were reported; the ‘shear-sandwich’ clamps were employed to evaluate the frequency-dependent shear modulus, in oscillation–frequency sweep mode, at a constant strain of 10%, with a logarithmic increase in the frequency from 0.1 to 10 Hz.

The reflectance spectra of the obtained hydrogels were registered using a GBC Cintra 303 spectrophotometer equipped with an integrating sphere at normal incidence, a band width of 0.42 nm, and a scanning speed of 1000 nm/min.

For the evaluation of the decontamination and CWA degradation studies, a Thermo Scientific Trace 1310 gas chromatograph and a TSQ 9000 triple quadrupole mass spectrometer (MS/MS) were used for the GC–MS experiments, utilizing a TR5MS GOLD capillary column, with the following conditions: carrier gas, helium, 1.5 mL/min; injection mode, splitless; injector temperature, 250 °C; temperature ramp, 40–300 °C; and heating rate, 10 °C/min. Using the MS/EI fragmentation, the toxic compounds and their degradation products were identified. Also, using the peak area the concentration of each component was established. Triplicate measurements were performed, and the mean values were reported.

## Figures and Tables

**Table 1:** Tensile stress–strain results for the ionic crosslinked hydrogels.

Sample	Stress (Pa) at 30% Strain	Maximum Stress (Pa)	Maximum Strain (%)
Alg-1	62,500	95,200	66.2
Alg-2	50,700	72,400	52.4
Alg-3	51,300	120,200	101.7
Alg-4c1	68,000	144,700	79.5
Alg-4c2	71,200	132,000	68.9
Alg-5	76,600	142,500	71.5

**Table 2:** Uniaxial compressive stress test results for the obtained hydrogels.

Sample	Stress (Pa) at 20% Strain	Stress (Pa) at 30% Strain	Maximum Stress (Pa)	Maximum Strain (%)
Alg-1	260,000	42,200	218,900	62.3
Alg-2	35,000	56,800	289,700	59.2
Alg-3	35,300	64,300	260,500	53.3
Alg-4c1	33,300	69,100	248,400	49.9
Alg-4c2	20,700	35,800	214,300	61.3
Alg-5	19,100	32,700	396,500	69.6

**Table 3:** Comparative decontamination/degradation efficacy overview.

Main Characteristics of the Decontaminating Formulation	Type of Contaminated Surface	Sulfur Mustard Entrapping Efficacy *	Degraded Sulfur Mustard	The Time Necessary for the Completion of the Decontamination Process	Refs.
An aqueous solution of commercial oxidizer (Oxone^TM^) and fumed silica	Acrylic painted metal	N/A (Not available)	50%	30 min	[43]
Chemical Decontamination Solution (CDS): hydrogen peroxide and sodium hydroxide	Aluminium	N/A	99%	60 min	[44]
	Chemical Agent Resistant Coating painted aluminum	N/A	97%	60 min	
EasyDECON^®^ DF200 (Surfactant, Peroxide, and Booster)	Glass	57%	N/A	30 min	[45]
	Concrete	23%	N/A	30 min	
	Galvanized metal	no significant effect	N/A	30 min	
BODIPY-embedded polymer coatings and fabrics for photocatalytic oxidation of sulfur mustard simulant CEES	PMMA	N/A	57%	60 min	[46]
	PS	N/A	60%	60 min	
	Army combat uniforms	N/A	30–100%	20–120 min	
Dry Reactive Polymers: chlorinated poly (4-vinylbenzenesulfonamide-co-methyl methacrylate) (PVMCl)	Polymeric substrate obtained via electrospinning	N/A	95%	60 min	[47]
Bentonite-supported silver nanoparticles dispersed in PVA/glycerol aqueous solution (film-forming)	Metallic surface	90.89%	N/A	20 h	[11]
Photocrosslinkable IPN hydrogel peelable films containing TiO_2_	Metallic surface	99.97	94.9%	120 min	[15]
Ionic crosslinked hydrogel films (eco-friendly, water-based, hydrolytic and photocatalytic effects, fast and efficient HD decontamination, peelable, safe, and easily disposable)	Metallic surface	92.3%	90.9%	35 min	This study

* According to the AEP—58 NATO standard, after decontamination, the residual sulfur mustard should not exceed [48] 0.1 mg/10 cm^2^.

**Table 4:** Composition of the decontaminating formulations.

Sample Code	Composition of the Hydrogels					
	PVA	Alg (Na)	BT	TiO_2_	PC-Ni	H_2_O
	wt.%	wt.%	wt.%	wt.%	wt.%	wt.%
Alg-1	2	2	-	-	-	96
Alg-2	2	2	0.5	-	-	95.5
Alg-3	2	2	0.5	0.1	-	94.8
Alg-4c1	2	2	0.5	-	0.005	95.5
Alg-4c2	2	2	0.5	-	0.01	95.5
Alg-5	2	2	0.5	0.1	0.005	95.4

## Data Availability

The original contributions presented in the study are included in the article/Supplementary Material, further inquiries can be directed to the corresponding author.

## Supplementary Materials

The following supporting information can be downloaded at: https://www.mdpi.com/article/10.3390/gels10070428/s1. Figure S1—SEM and EDX spectra of the obtained gels; Figure S2—SEM-EDX elemental mapping of sample Alg-5; Figure S3—Images during the deposition of the decontamination solutions and peeling of the crosslinked hydrogels; Figure S4—Images of the gels during the mechanical tests; Figure S5—Images of the gels during swelling degree assessments experiments; Figure S6—UV-Vis spectra of the hydrogels Alg-3, Alg-4c1, Alg-4c2, and Alg-5 (A-reflectance; B-absorption) and Tauc plots (C); Figure S7—Chromatograms for exemplifications—determination of non-degraded HD concentrations entrapped in the hydrogels; Figure S8—Proposed mechanism for the HD degradation in the hydrogels in the presence of photocatalytic components (TiO_2_ and/or NiPc) and light (blue—hydrolytic steps; green—photoinduced oxidative steps).
