# Supplementary material for: Ionic Crosslinked Hydrogel Films for Immediate Decontamination of Chemical Warfare Agents
Source: Gels. 2024 Jun 28;10(7):428. doi: 10.3390/gels10070428 (PMC11275507; doi:10.3390/gels10070428)
Supplement: Supplementary file 1 [file gels-10-00428-s001.zip › gels-3056570-supplementary.pdf]

Ionic Crosslinked Hydrogel Films for Immediate Decontamination of Chemical Warfare Agents

Gabriela Toader [1,†], Raluca-Elena Ginghina [2,†], Adriana Elena Bratu [2,3], Alice Ionela Podaru [1], Daniela Pulpea [1], Traian Rotariu [1], Ana Mihaela Gavrilă [4] and Aurel Diacon [1,3,*]

[1] Military Technical Academy 'Ferdinand I', 39–49 George Coșbuc Blvd., 050141 Bucharest, Romania; gabriela.toader@mta.ro (G.T.); podaru.alice04@gmail.com (A.I.P.); daniela.pulpea@mta.ro (D.P.); traian.rotariu@mta.ro (T.R.)
[2] Research and Innovation Center for CBRN Defense and Ecology, 225 Olteniței Blvd., 077160 Bucharest, Romania; raluca.ginghina@nbce.ro (R.-E.G.); adriana.bratu@nbce.ro (A.E.B.)
[3] Faculty of Chemical Engineering and Biotechnologies, National University of Science and Technology Politehnica of Bucharest, 1–7 Gh. Polizu Street, 011061 Bucharest, Romania
[4] National Institute of Research and Development for Chemistry and Petrochemistry, 202 Splaiul Independentei, 060041 Bucharest, Romania; ana.gavrila@icechim.ro
* Correspondence: aurel.diacon@mta.ro
† These authors contributed equally to this work.

Table of Contents

Figure S1. SEM and EDX spectra of the obtained hydrogels films 7

Figure S2. SEM-EDX elemental mapping of sample Alg-5 8

Figure S3. Images during the deposition of the decontamination solutions and peeling of the crosslinked hydrogels 9

Figure S4. Images of the gels during the mechanical tests 10

Figure S5. Images of the gels during swelling degree assessment experiments 11

Figure S6. UV-Vis spectra of the hydrogels Alg-3, Alg-4c1, Alg-4c2 and Alg-5 (A-reflectance; B-absorption) and Tauc plots (C) 12

Figure S7. Chromatograms for exemplifications - determination of non-degraded HD concentrations entrapped in the hydrogels 13

Figure S8. Proposed mechanism for the HD degradation in the hydrogels in the presence of photocatalytic components (TiO_2 and/or NiPc) and light (blue – hydrolytic steps; green – photoinduced oxidative steps) 14

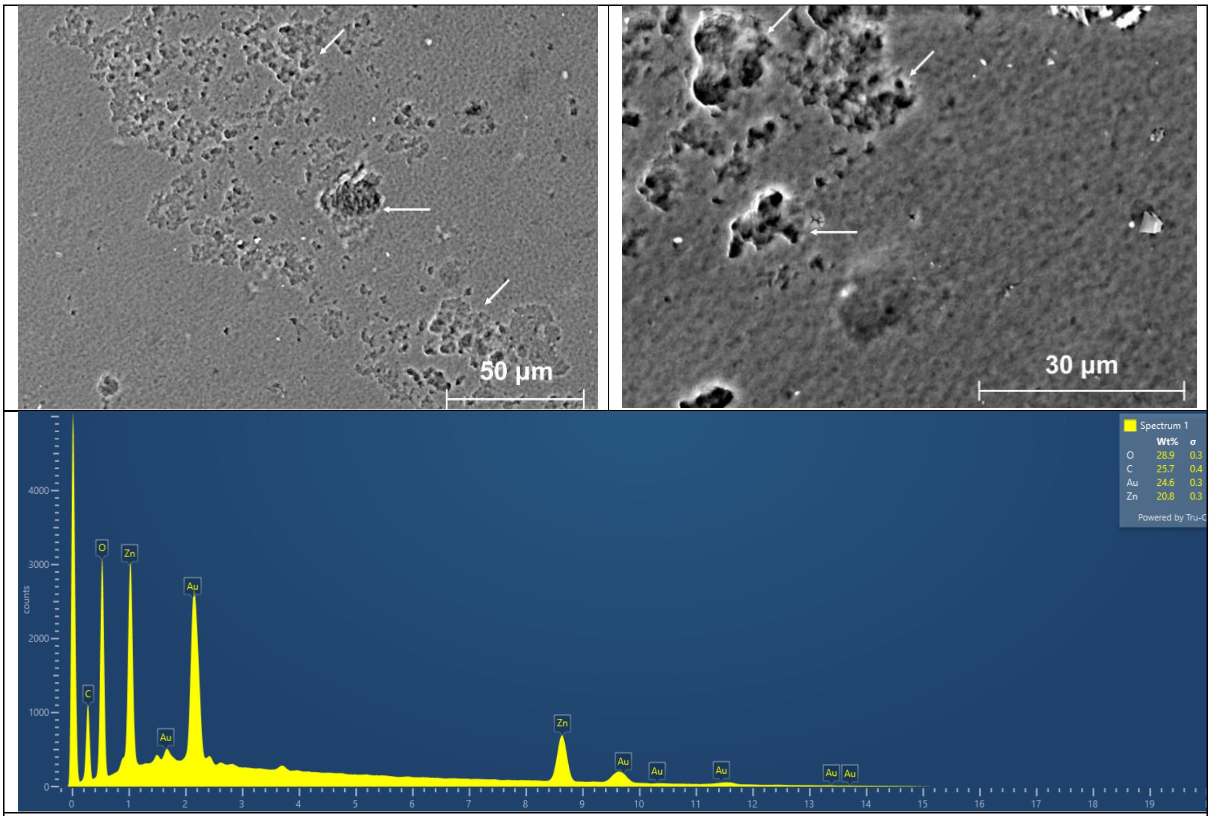
50 µm
30 µm
Spectrum 1
Wt% σ
O 28.9 0.3
C 25.7 0.4
Au 24.6 0.3
Zn 20.8 0.3
counts
C
O
Zn
Au
Au
Zn
Au
Au
Au
Au
Au

Alg-1

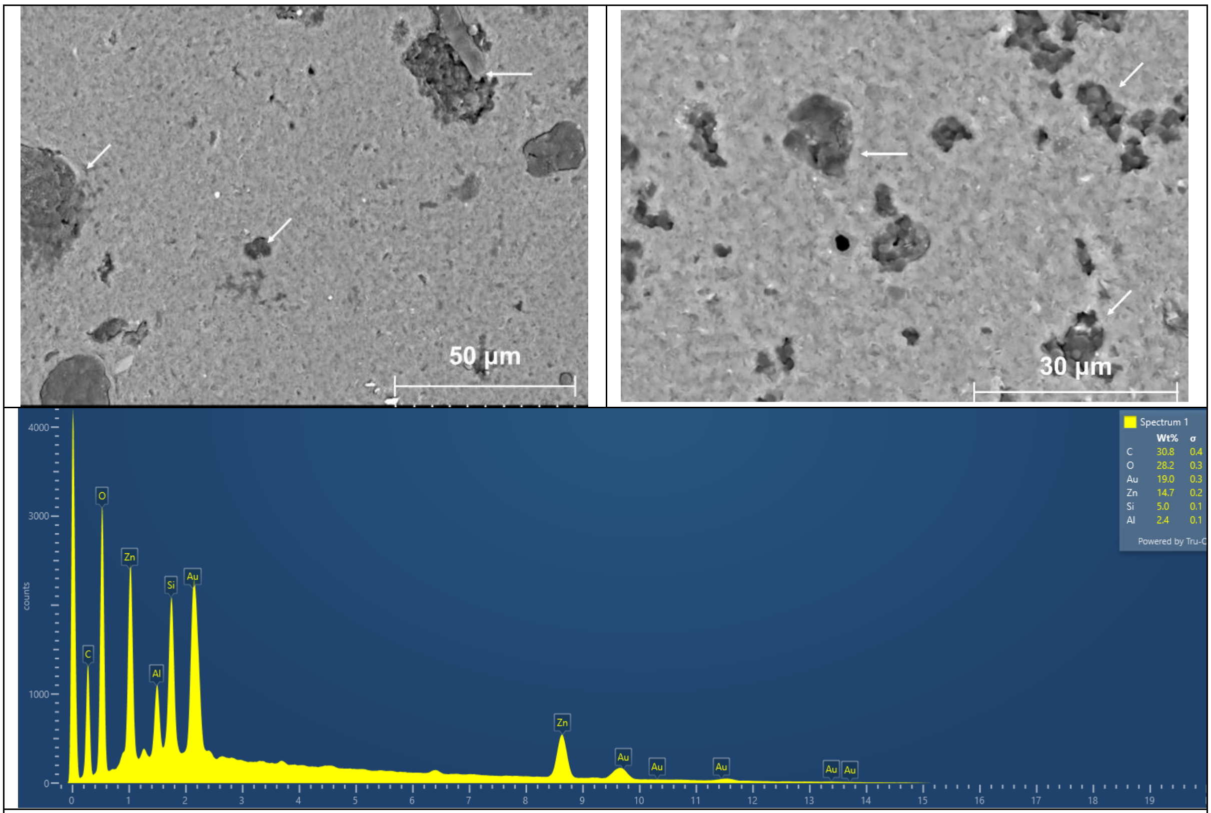
50 µm
30 µm
Spectrum 1
Wt% σ
C 30.8 0.4
O 28.2 0.3
Au 19.0 0.3
Zn 14.7 0.2
Si 5.0 0.1
Al 2.4 0.1
Powered by Tru-Q
counts
4000
3000
1000
0
C
O
Zn
Si
Au
Al
Zn
Au
Au
Au
Au Au
0 1 2 3 4 5 6 7 8 9 10 11 12 13 14 15 16 17 18 19

Alg-2

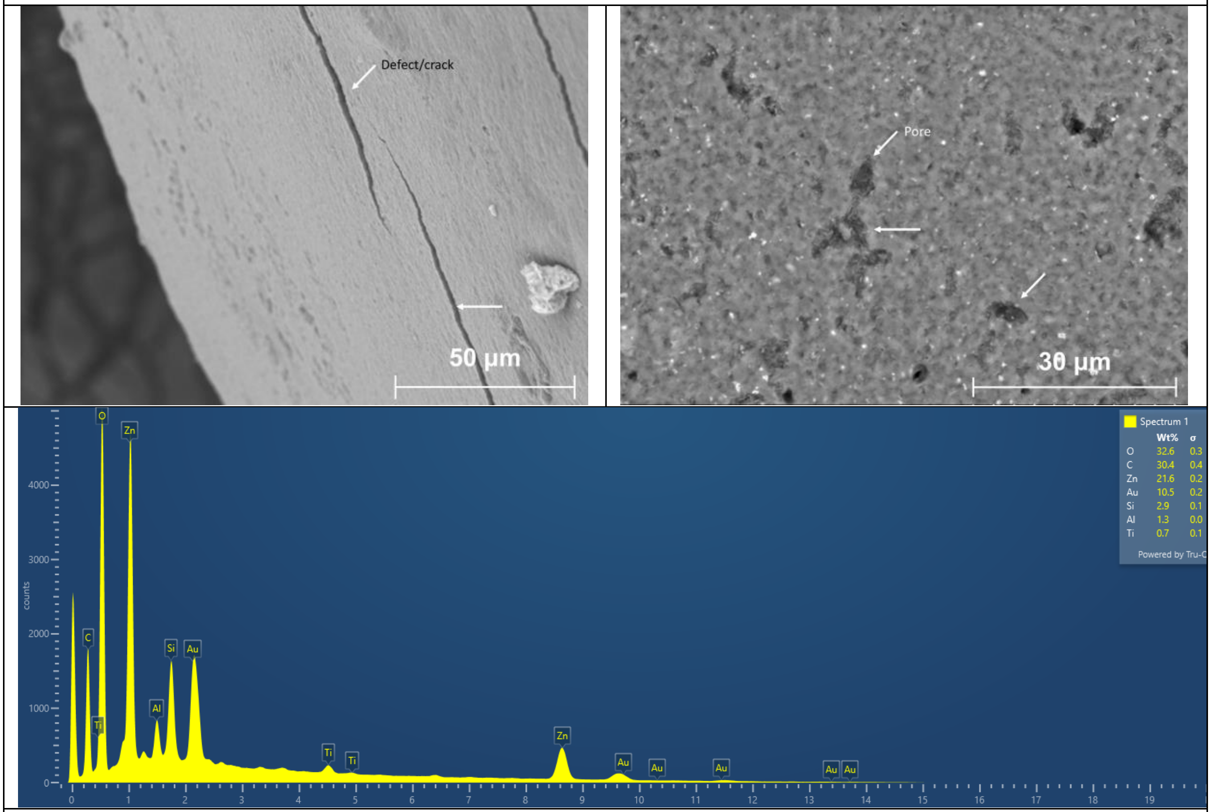
Defect/crack
50 µm
Pore
30 µm
counts
Spectrum 1
Wt% σ
O 32.6 0.3
C 30.4 0.4
Zn 21.6 0.2
Au 10.5 0.2
Si 2.9 0.1
Al 1.3 0.0
Ti 0.7 0.1
Powered by Tru-Q

Alg-3

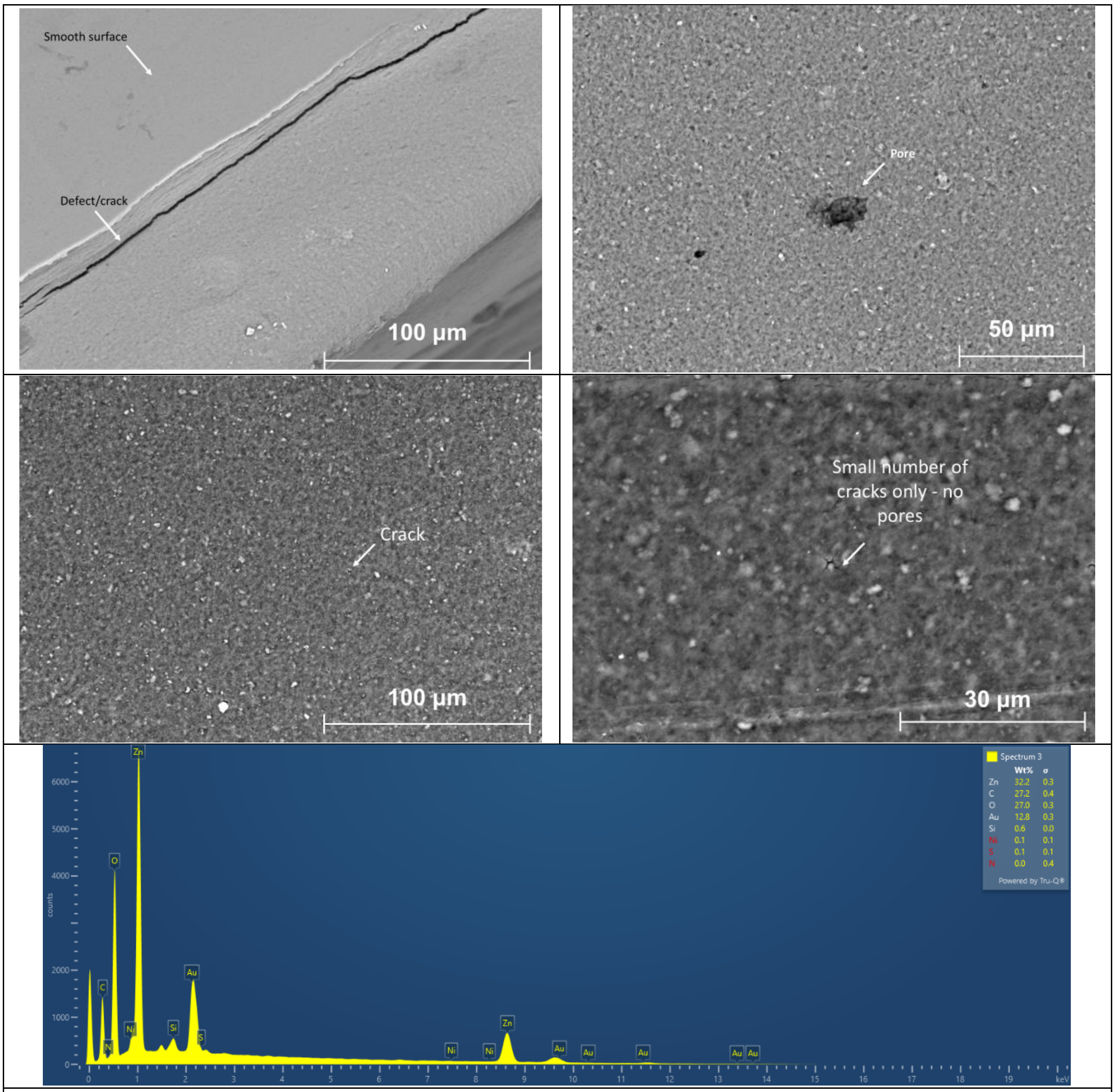
Smooth surface
Defect/crack
100 µm
Pore
50 µm
Crack
100 µm
Small number of cracks only - no pores
30 µm
Spectrum 3
Wt% σ
Zn 32.2 0.3
C 27.2 0.4
O 27.0 0.3
Au 12.8 0.3
Si 0.6 0.0
Ni 0.1 0.1
S 0.1 0.1
N 0.0 0.4
Powered by Tru-Q®
counts
keV

Alg-4c1

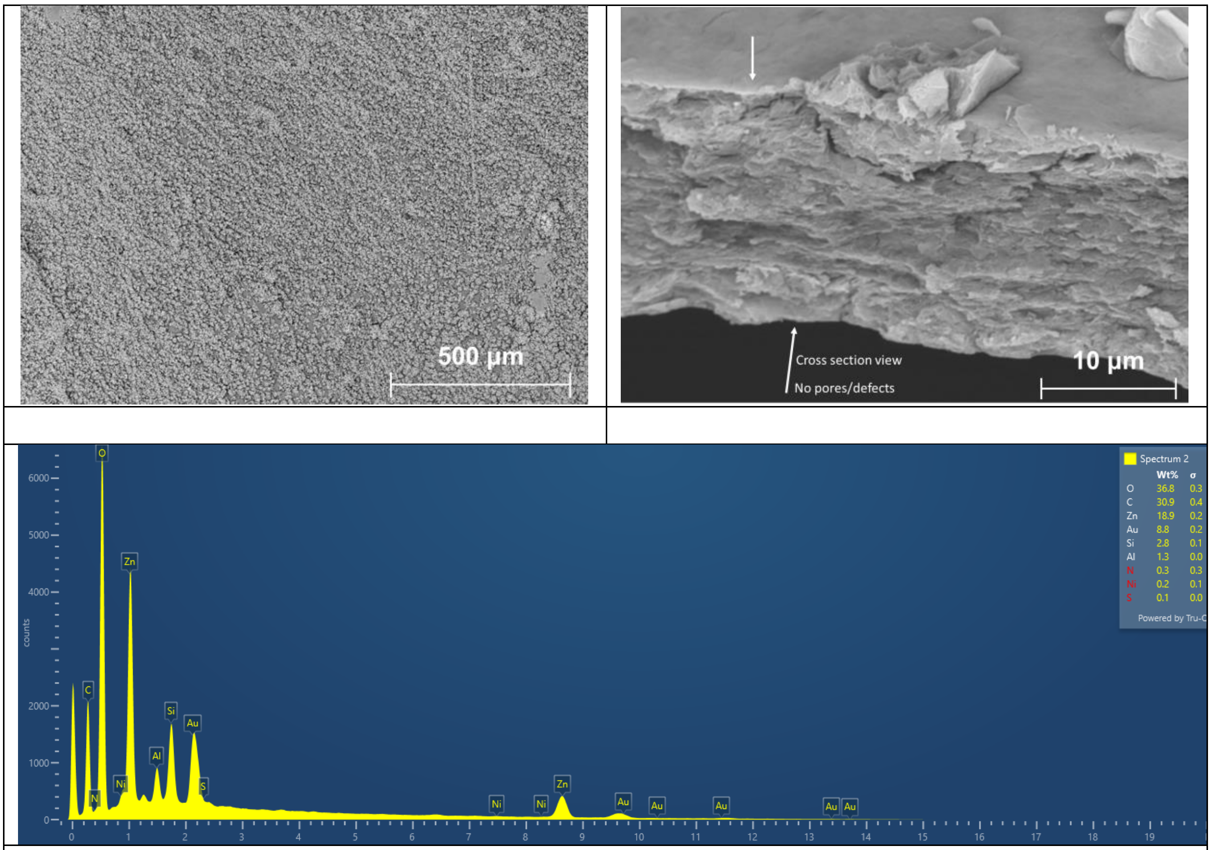
500 µm
Cross section view
No pores/defects
10 µm
Spectrum 2
Wt% σ
O 36.8 0.3
C 30.9 0.4
Zn 18.9 0.2
Au 8.8 0.2
Si 2.8 0.1
Al 1.3 0.0
N 0.3 0.3
Ni 0.2 0.1
S 0.1 0.0
Powered by Tru-C
counts
O
Zn
C
Si
Au
Al
Ni
S
Ni
Ni
Zn
Au
Au
Au
Au
Au

Alg-4c2

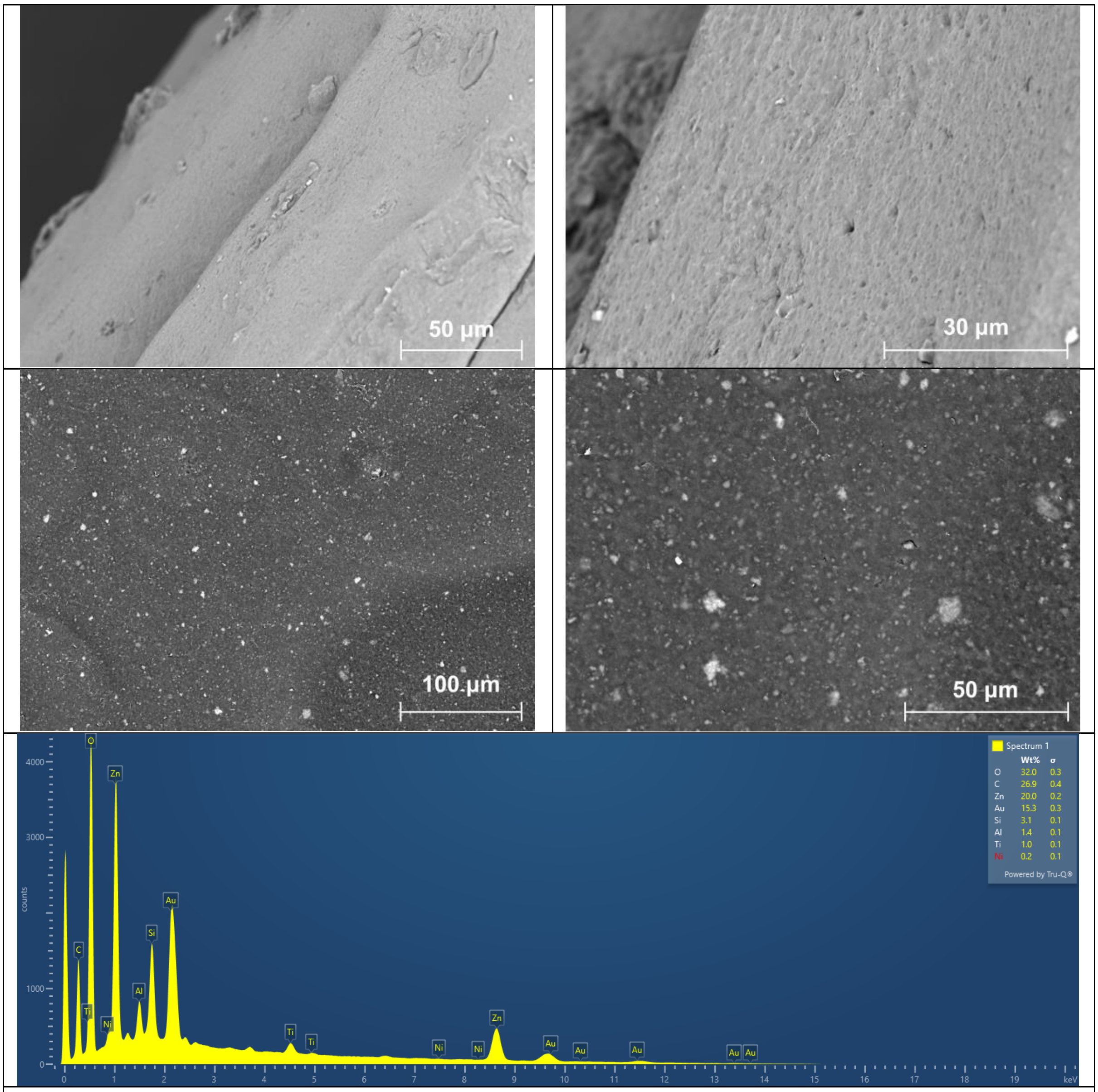

Alg-5

Figure S1. SEM and EDX spectra of the obtained hydrogels films

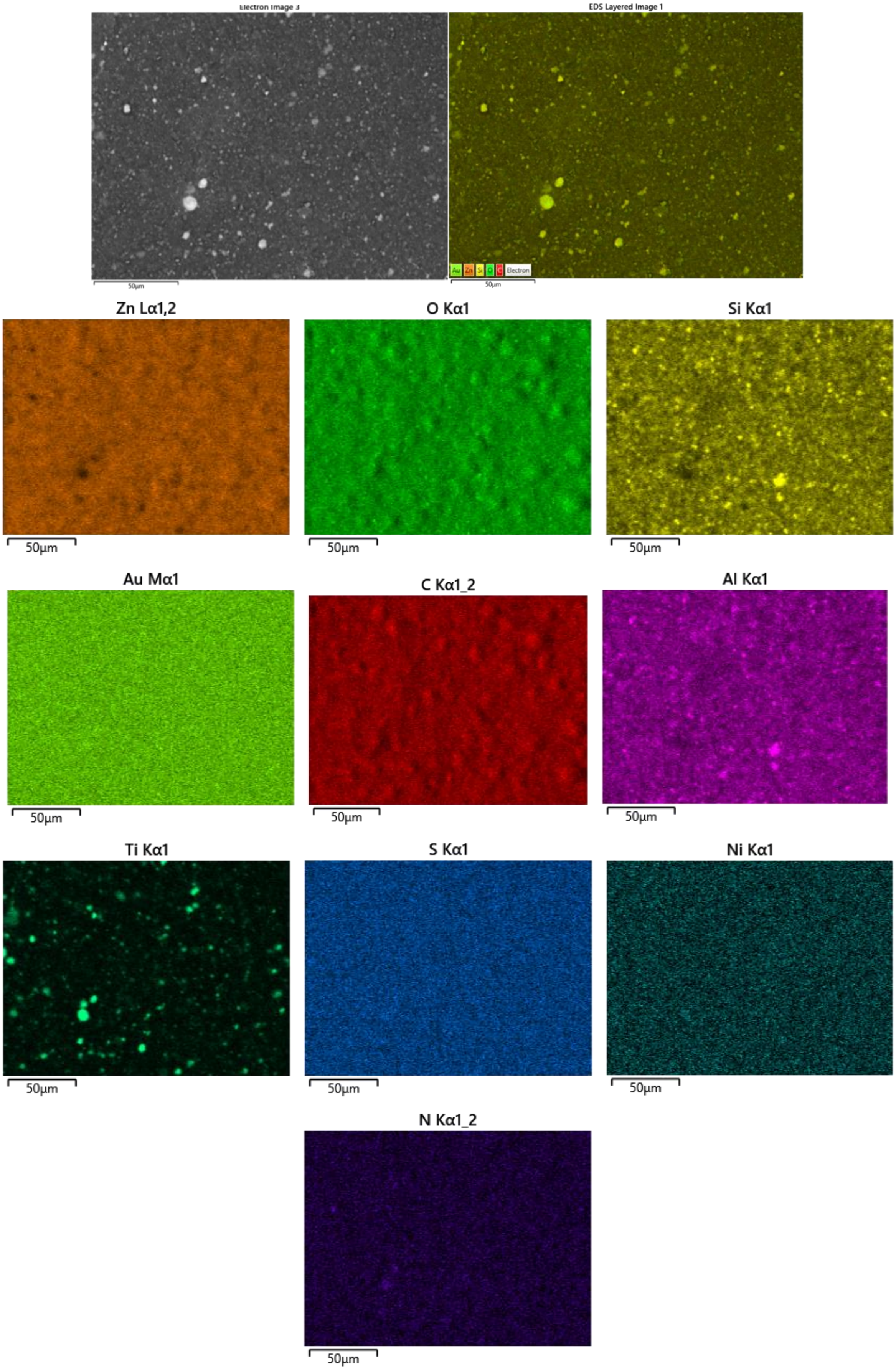

Figure S2. SEM-EDX elemental mapping of sample Alg-5

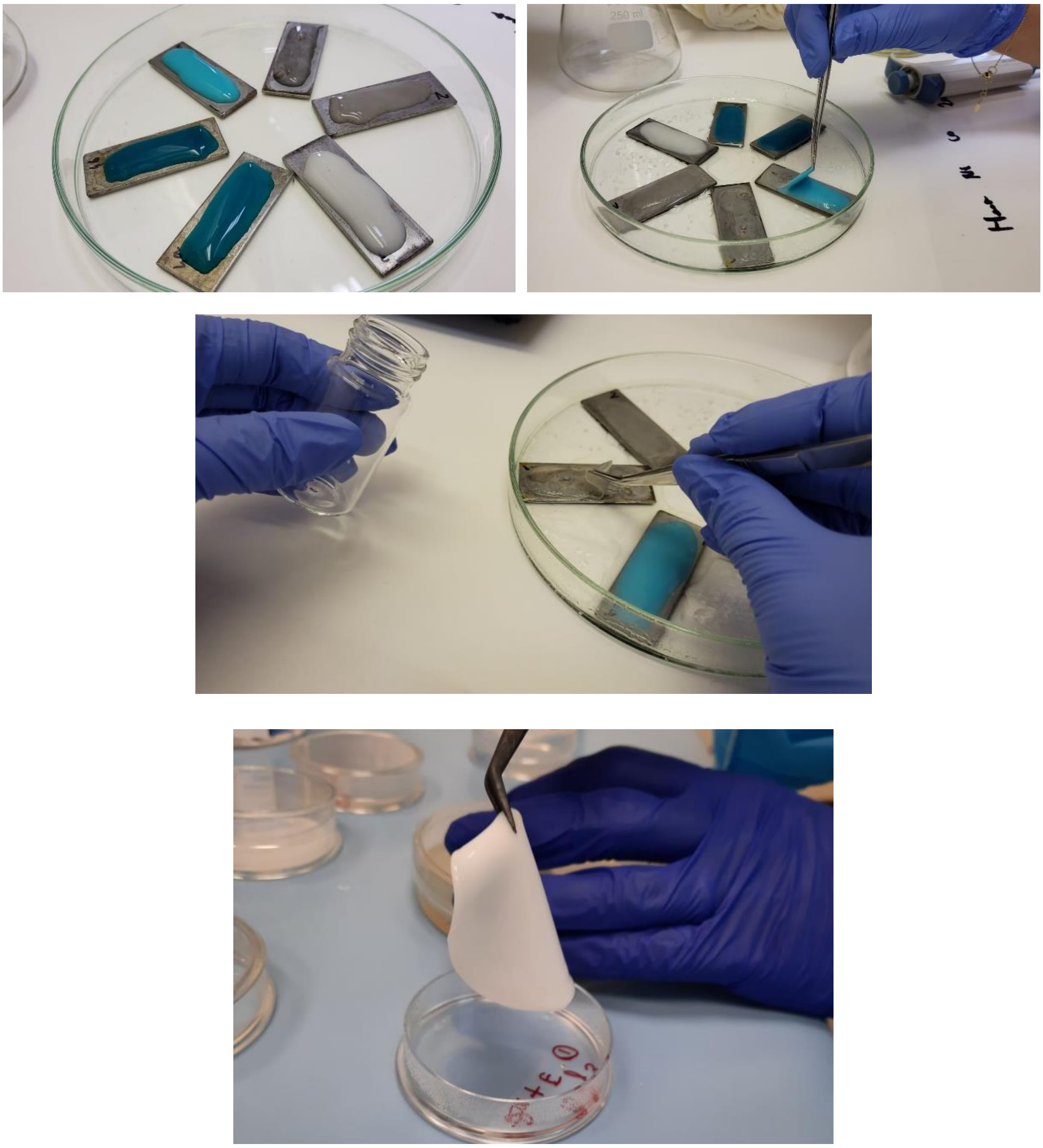

Figure S3. *Images during the deposition of the decontamination solutions and peeling of the crosslinked hydrogels*

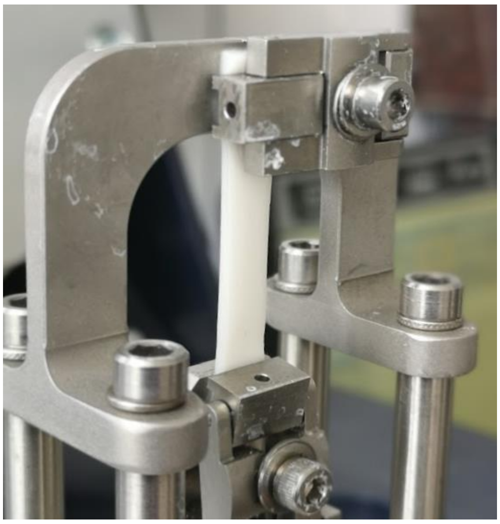

Alg-3 – Tensile tests

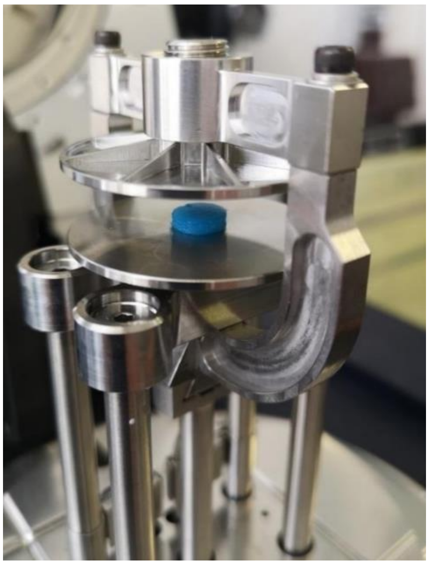

Alg-5 - Compression

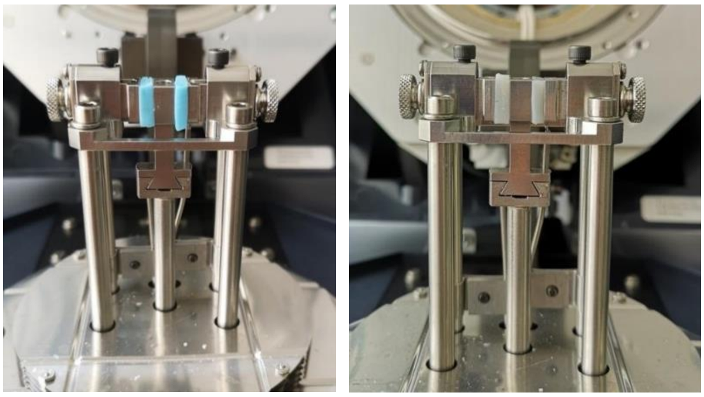

Alg-5 – Shear test

Figure S4. *Images of the gels during the mechanical tests*

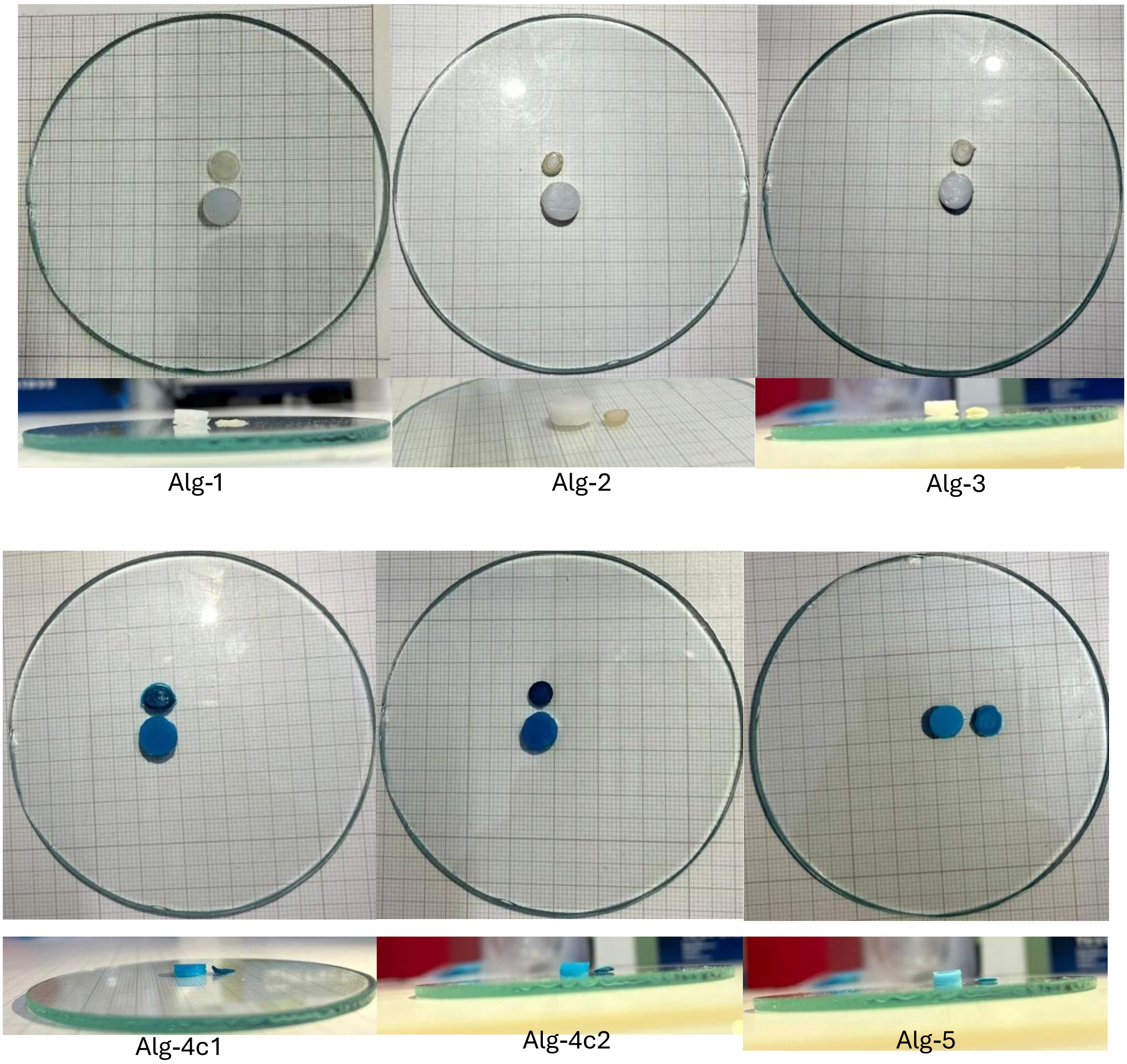

Figure S5. *Images of the gels during swelling degree assessment experiments*

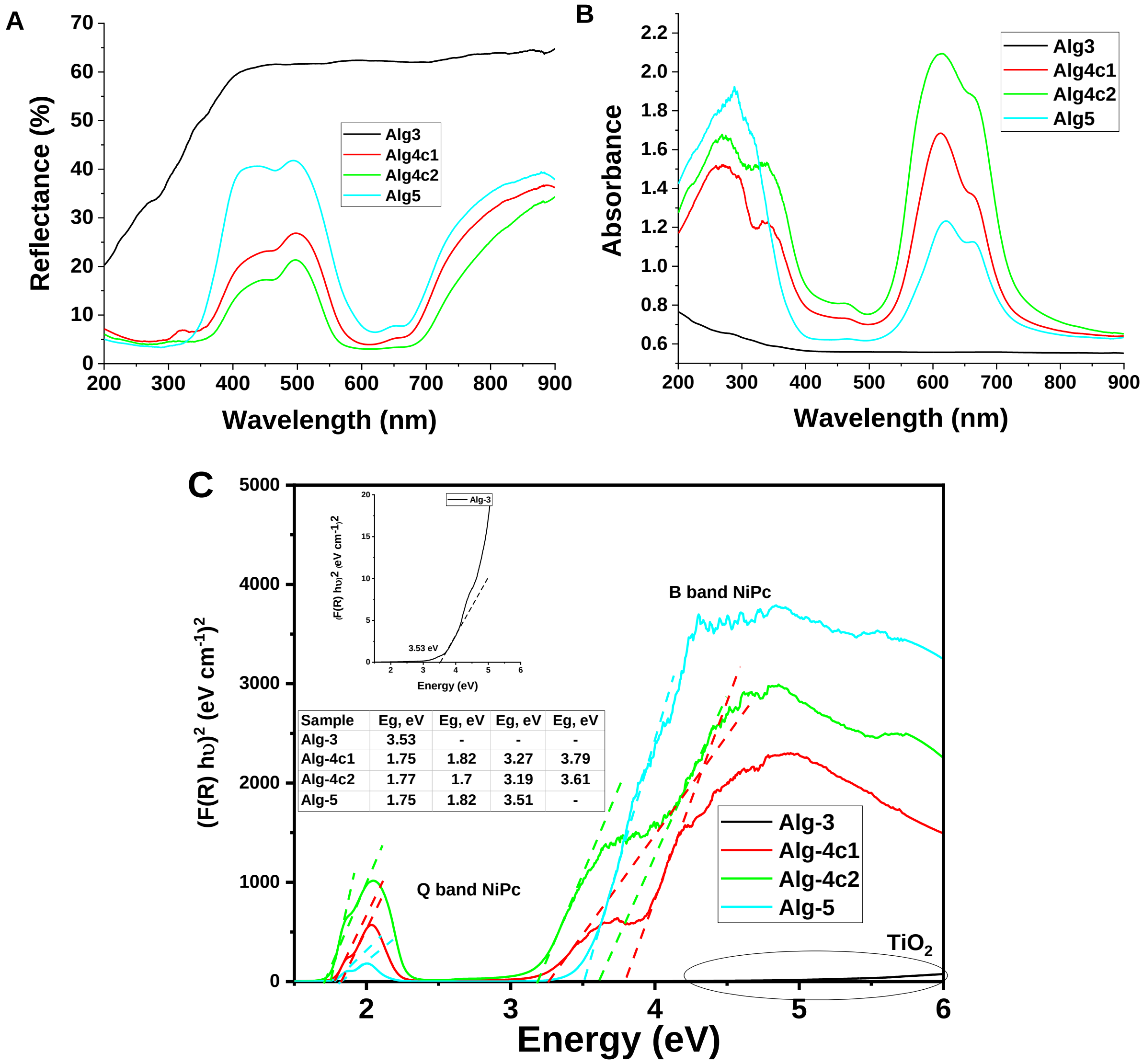

Sample	Eg, eV	Eg, eV	Eg, eV	Eg, eV
Alg-3	3.53	-	-	-
Alg-4c1	1.75	1.82	3.27	3.79
Alg-4c2	1.77	1.7	3.19	3.61
Alg-5	1.75	1.82	3.51	-

Figure S6. *UV-Vis spectra of the hydrogels Alg-3, Alg-4c1, Alg-4c2 and Alg-5 (A-reflectance; B-absorption) and Tauc plots (C)*

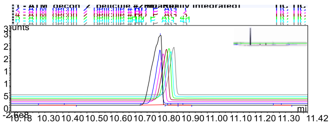

Figure S7. *Chromatograms for exemplifications - determination of non-degraded HD concentrations entrapped in the hydrogels*

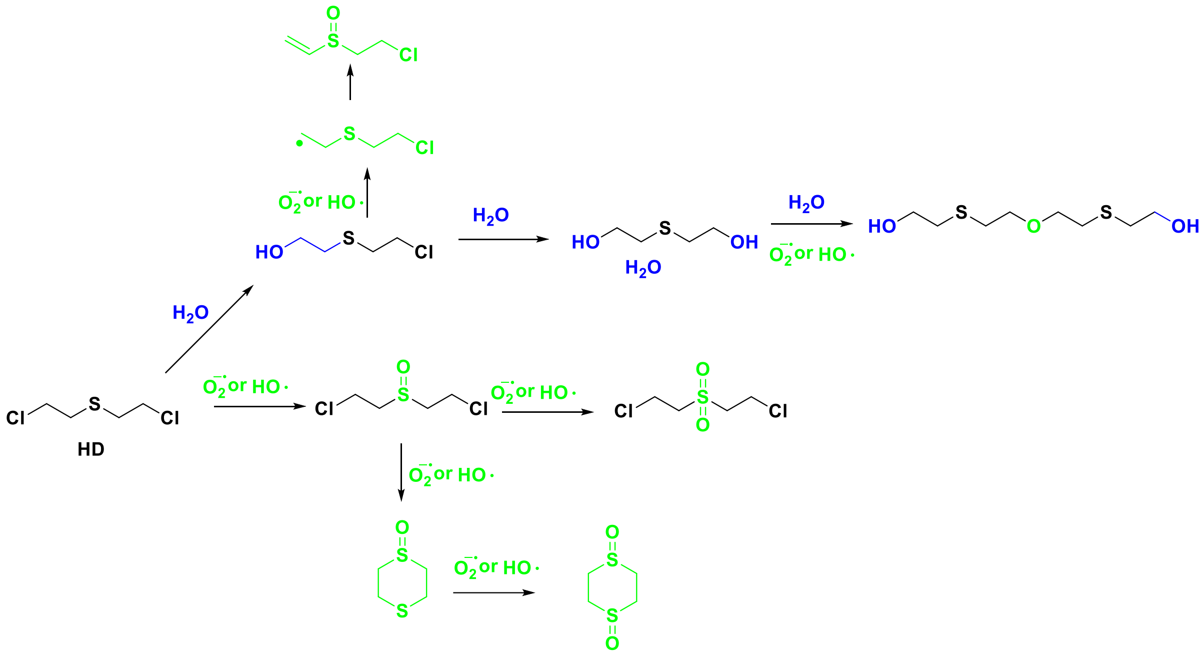

Figure S8. *Proposed mechanism for the HD degradation in the hydrogels in the presence of photocatalytic components (TiO_2 and/or NiPc) and light (blue – hydrolytic steps; green – photoinduced oxidative steps)*
